# Vaginal Swabs Are Non-inferior to Endocervical Swabs for Sexually Transmitted Infection testing in the Emergency Department

**DOI:** 10.5811/westjem.2022.3.53812

**Published:** 2022-05-02

**Authors:** Andrew Krause, Joseph B. Miller, Linoj Samuel, Jacob J. Manteuffel

**Affiliations:** * Henry Ford Hospital, Department of Emergency Medicine, Detroit, Michigan; † Henry Ford Hospital, Department of Pathology, Detroit, Michigan

## Abstract

**Study Objective:**

Emergency department (ED) testing for sexually transmitted infections (STI) in women is typically performed with a pelvic examination and an endocervical swab. However, vaginal swabs are effective for STI testing and the preferred specimen type according to the US Centers for Disease Control and Prevention. The utility of using vaginal swabs in the ED for STI screening has not been thoroughly investigated. Our objective was to assess detection rates for two bacterial STIs before and after implementing a screening protocol using vaginal swabs.

**Methods:**

We conducted a quasi-experimental, pre-post study using standardized data from electronic health records across nine metropolitan Detroit hospital EDs. Patients included women who were tested for *Chlamydia trachomatis* or *Neisseria gonorrhoeae* in the ED between April 2018–December 2019. Pre-implementation tests from April 2018–February 2019 were done using endo-cervical swabs, and post-implementation tests from February 2019–December 2019 were done with vaginal swabs. We used non-inferiority testing for proportion with a non-inferiority margin of one percentage point absolute difference in detection rates of STI.

**Results:**

The study included 22,291 encounters with 11,732 in the pre-implementation and 10,559 in the post-implementation phases. The *C. trachomatis* detection rates were 7.5% pre-implementation and 7.6% post-implementation (between-group difference, 0.1 percentage points; 95% confidence interval [CI]: −0.7, 0.4; p<.01 for non-inferiority). The *N. gonorrhoeae* detection rates were 3.1% pre-implementation and 3.6% post-implementation (between-group difference, 0.5 percentage points; 95% CI: −0.8, 0.04; p<.01 for non-inferiority).

**Conclusion:**

Using vaginal swabs for STI testing in the ED may be a non-inferior alternative to using endocervical swabs.

## INTRODUCTION

### Background

Treatment for newly acquired sexually transmitted infections (STI) was estimated to cost $16 billion in the United States in 2018, with the treatment of *Chlamydia trachomatis* and *Neisseria gonorrhoeae* totaling almost $100 million.^1^ The US Centers for Disease Control and Prevention (CDC) reported that *C trachomatis* rates in the US increased by 11.4% in women from 2014 to 2018, while *N. gonorrhoeae* infection rates in women increased by 3.6% from 2017 to 2018.^2^ Infection with these organisms puts women at risk for infertility, ectopic pregnancy, pelvic inflammatory disease, and increased risk for human immunodeficiency virus infection.^3^ Expanding screening approaches may help to reduce STI rates.

The CDC recommends vaginal swabs as an appropriate sample type when testing for many STIs, even when a pelvic exam is performed.^4^ The US Food and Drug Administration has approved both endocervical and intravaginal swabs as screening methods for *C. trachomatis* and *N. gonorrhoeae.*^5^ For STI screening in women, self-collected vaginal swabs have been shown to have a similar sensitivity and specificity to those collected by a clinician.^6^ Endocervical samples for *C. trachomatis* testing in young adult women have shown sensitivities ranging from 75–100%, with some reports of sensitivities greater than 90%.^4^,^7^ Self-collected vaginal swabs from young women have shown nucleic acid amplification technique sensitivities ranging from 75–100% for the detection of *C. trachomatis*.^7^ In addition, results from nucleic acid amplification technique tests for *N. gonorrhoeae* have been reported to be similar to those for *C trachomatis*, with endocervical sample sensitivities of 89–97% and vaginal sample sensitivities of greater than 90%.^4^,^7^ Other studies have even found that *C. trachomatis* and *N. gonorrhoeae* detection rates are higher from patient-performed vaginal swabs than from endocervical swabs.^7^

### Importance

Few studies have investigated the use of vaginal swabs in the emergency department (ED) for STI testing.^8^ One small previous study demonstrated that patient self-collected vaginal swabs were not inferior for detecting *C. trachomatis* and *N. gonorrhoeae* in a single ED.^9^ The use of vaginal swabs for STI testing in the ED provides an option for a patient-collected sample in appropriate situations. This can reduce the need for the more invasive procedure of a pelvic exam, saving time and resources, and perhaps promoting patient autonomy and reducing patient stress.

### Goals of This Investigation

We implemented a protocol for using vaginal swabs, rather than endocervical swabs, to test for two bacterial STIs in women in EDs within a multihospital health system. We hypothesized that STI detection rates using vaginal swabs in the ED would be equivalent to the pre-implementation protocol that used only endo-cervical swabs collected by clnicians.

## METHODS

### Ethics Approval

Approval for this study was obtained from the Henry Ford Health System Institutional Review Board prior to the start of this study.

### Study Design and Setting

This was a quasi-experimental, retrospective pre-post study using standardized data from the electronic health record (EHR) across the Henry Ford Health System. We assessed 10 months of data before (pre-implementation phase from April 2018–February 2019) and 10 months of data after (post-implementation phase from February 2019–December 2019) the implementation of an ED STI vaginal swab screening intervention. The study period included records of STI tests that were done in nine EDs in the metropolitan Detroit, Michigan, area. Eligible patients included any woman who received testing for *C. trachomatis* or *N. gonorrhoeae* in the nine EDs during the study period. Retrospective chart review for data collection was obtained following recommendations by Worster et al to reduce bias and adhere to methodologic standards for medical record review.^10^

Because this was a retrospective study clinicians were blinded to the study during the data collection period. Abstractors were properly trained on data collection and analysis for data from our EHR system prior to analysis. Multiple trained abstractors reviewed the same data to ensure data results were correct and accurate. Abstractors were blinded to the goals of the study during the analysis stage of this study. We excluded duplicate patient visits from data analysis. If data was missing from a specific patient, that patient was removed from the data set prior to analysis.

### Selection of Participants

Women included in this study were in the ED for symptomatic STIs such as pelvic pain, unusual or foul-smelling discharge, or lower abdominal pain, had screened positive for possible STI exposure during history-taking, or were asymptomatic but had asked to be tested for STIs. Pregnant patients or patients with vaginal bleeding were not excluded from data collection. Men were excluded from this study.

### Interventions

The intervention consisted of a new ED protocol that introduced vaginal swabs to test for STIs rather than endocervical swabs. In the pre-implementation phase, endocervical swabs were collected using the Aptima Unisex Swab Specimen Collection Kit (Hologic, Inc., Marlborough, MA). At the time of the intervention, use of endocervical swabs was discontinued in all nine EDs and only vaginal swabs were available for testing. All patients who would have been previously swabbed using the endocervical swabs were swabbed using intravaginal swabs in the post-implementation phase. Following Michigan Department of Health and Human Services (MDHHS) and CDC policies, if patients reported no unusual or foul-smelling discharge, pelvic pain, or dyspareunia, they were offered the opportunity to self-collect the swab during the post-implementation phase.^11^

In the post-implementation phase, swabs were collected by clinicians or self-collected by the patient. Clinicians would perform the vaginal swabs if the patient had any of the above symptoms, if asymptomatic patients requested that the clinician collect the swabs, or if the patient was unable to perform the swab herself. Patients who collected their own vaginal swabs were provided instructions on how to perform the intravaginal swabs per MDHHS and CDC policies prior to collection by either a nurse or clinician. The intravaginal swabs were collected by carefully introducing the swab about two inches past the introitus. The swab was moved circumferentially around the intravaginal canal for 10–30 seconds. Special attention was made to make sure the swab touched the walls of the vagina and absorbed the moisture. The swab was then directly placed in the collection tube and sent to the lab for analysis.

Specimens for testing for *N. gonorrhoeae* and *C. trachomatis* were collected using the Aptima Vaginal Swab Specimen Collection Kit (Hologic) following manufacturer’s instructions. Testing for *N. gonorrhoeae* and *C. trachomatis* was performed by transcription-mediated amplification using the Aptima Combo 2 assay on the Panther platform (Hologic).

### Measurements and Outcomes

Patients were considered to have an STI only if the laboratory results from the STI screening for *C. trachomatis* and *N. gonorrhoeae* result were positive.

### Analysis

We estimated the requisite sample size to be 19,770 encounters with laboratory test results to ensure a non-inferiority margin of one percentage point absolute difference in detection rates of STIs. This estimate assumed a power of 95% and alpha = 0.05. Analysis consisted of non-inferiority testing for proportion with a non-inferiority margin of one percentage point absolute difference in detection rates of STI. Only patients with definite positive or negative result were included in the data analysis. Equivocal test results were excluded from the data collection. We completed analysis with SAS 9.4 (SAS Institute, Inc., Cary, NC). We report between-group differences with their associated 95% confidence interval (CI).

## RESULTS

The study included 22,291 encounters across nine EDs within one multihospital health system. A total of 11,732 encounters occurred during the pre-implementation phase and 10,559 occurred in the post-implementation phase. The post-implementation group included intravaginal swabs that were performed either by clinicians or were self-swabbed by the patient, while pre-implementation tests used endocervical swabs collected solely by clinicians. The rate of detection of *C. trachomatis* was 7.5% pre-implementation and 7.6% post-implementation (between-group difference, 0.1 percentage points; 95% CI: −0.7, 0.4; *P* <.01 for non-inferiority). The rate of detection of *N. gonorrhoeae* was 3.1% pre-implementation and 3.6% post-implemen-tation (between-group difference, 0.5 percentage points; 95% CI: −0.8, 0.04; *P* < 0.1 for non-inferiority). Data is listed in the Table. In Wayne County, Michigan, there were 1330.4 cases/month of *C. trachomatis* in the pre-implementation period and 1284.5 cases/month in the post-implementation period. There were 583.6 cases/month of *N. gonorrhoeae* in the pre-implementation period and 595.3 cases/month in the post-implementation period; therefore, there was not a significant temporal change in infection case rates between the two time periods.^12^

## DISCUSSION

In this retrospective pre-post study, we showed that using vaginal swabs for STI testing in the ED resulted in *C. trachomatis* and *N. gonorrhoeae* detection rates similar to those from using endocervical swabs. There have been limited studies assessing the utility of patient-administered vaginal swabs for STI testing in the ED; however, some studies have shown comparable or even higher sensitivities from using intravaginal swabs compared to endocervical swabs.^6^,^7^,^9^

Allowing an asymptomatic patient to collect her own vaginal swab for STI testing limits the demands on resources and personnel in the ED, since a traditional pelvic exam can be time and resource intensive. Performing a pelvic exam often requires moving the patient to a pelvic exam room, cleaning an additional room, finding a chaperone for the patient, and adding time to the clinician’s workload. Avoiding these steps by having patients administer their own swabs can save considerable time and money. Previous studies have reported increased comfort among female patients who collect their own test samples compared to clinician- collected swabs.^13^ Patients may feel more comfortable collecting their own swabs and may be more open to being tested for STIs with this approach. This may lead to greater STI-detection rates.

In summary, the results of this study showed that detection rates of *C. trachomatis* and *N. gonorrhoeae* were non-inferior when a protocol allowing for intravaginal swabs was introduced into multiple EDs within a single healthcare system. Future research could include an ED-specific patient questionnaire to determine whether self-collected swabs are viewed positively or negatively by patients in the ED. A follow-up study could evaluate clinician-collected vaginal swabs compared to patient-collected swabs to determine whether there is a difference in detection rates for *C. trachomatis* and *N. gonorrhoeae.* Additional studies might investigate whether there is a difference in detection rates for other STIs such as *Trichomonas vaginalis* or bacterial vaginosis.

## LIMITATIONS

Limitations of this study include inter-reliability of clinicians collecting true endocervical swabs vs possible intravaginal swabs in the pre-implementation study phase. Although clinicians and nurses gave patients instructions on how to perform the intravaginal swabs per MDHHS and CDC policies prior to collection in the post-implementation study phase, certain patients may not have collected true intravaginal swabs, which may have lowered the detection rates for STIs. Due to the retrospective nature of the study and absence of documentation, we were unable to extract whether a patient’s sample was self-collected. Most of our patients undergoing testing for STI are symptomatic requiring a pelvic exam, but the precise proportion of self-collected swabs is unknown. This was an observational study within a single healthcare system; thus, results are not generalizable.

## CONCLUSION

An ED protocol using vaginal swabs for *C. trachomatis* and *N. gonorrhoeae* testing for women may be non-inferior to the use of endocervical swabs for STI detection rates for these bacterial pathogens.

## Figures and Tables

**Table:** Detection rates for *Chlamydia trachomatis* and *Neisseria gonorrhoeae* infection in women before and after implementation of a testing program for sexually transmitted infections, using endocervical swabs vs vaginal swabs (N = 22,291).

	Pre-implementation infection rate (endocervical swab) (N = 11,732)	Post-implementation infection rate (vaginal swab) (N = 10,559)	Between-group difference	95% CI for non-inferiority	*P*-value
*C. trachomatis*	7.5% (N = 880)	7.6% (N = 802)	0.1%	(−0.7, 0.4)	<.01
*N. gonorrhoeae*	3.1% (N = 364)	3.6% (N = 380)	0.5%	(−0.8, 0.04)	<.01

*CI*, confidence interval.
